# Are Diet and Physical Activity Patterns Related to Cigarette Smoking in Adolescents? Findings From Project EAT

**Published:** 2007-06-15

**Authors:** Nicole I Larson, Mary Story, Dianne Neumark-Sztainer, Peter J Hannan, Cheryl L Perry

**Affiliations:** Division of Epidemiology and Community Health, School of Public Health, University of Minnesota; Division of Epidemiology and Community Health, School of Public Health, University of Minnesota, Minneapolis, Minn; Division of Epidemiology and Community Health, School of Public Health, University of Minnesota, Minneapolis, Minn; Division of Epidemiology and Community Health, School of Public Health, University of Minnesota, Minneapolis, Minn; School of Nursing, The University of Texas at Austin, Austin, Tex

## Abstract

**Introduction:**

An inadequate diet and physical inactivity may compound the many deleterious effects of smoking on health. Some research indicates that smoking behavior is related to other health behaviors, but little research has examined how smoking may be related to dietary intake of key nutrients, consumption of fast food, sedentary lifestyle, or weight status. The purpose of this study was to describe smoking frequency among adolescents and its relationship to physical activity and dietary patterns.

**Methods:**

The research study employed a cross-sectional, population-based design. Adolescents self-reported cigarette smoking, physical activity, and eating behaviors on the Project EAT (Eating Among Teens) survey and reported dietary intake on a food frequency questionnaire completed in school classrooms. The sample included 4746 middle school and high school students from Minneapolis-St. Paul public schools. Mixed-model regression, which was controlled for sex, race and ethnicity, socioeconomic status, grade level (middle school or high school), and school, was used to examine the association of smoking with diet and physical activity patterns.

**Results:**

Overall, reported smoking frequency was inversely related to participating in team sports, eating regular meals, and consuming healthful foods and nutrients. Smoking frequency was directly related to frequency of fast-food and soft drink consumption.

**Conclusion:**

Adolescents who smoke cigarettes may be less likely to engage in health-promoting lifestyle behaviors. Interventions are needed to prevent smoking and the unhealthy dietary practices and physical activity behaviors that may be associated with it.

## Introduction

Cigarette smoking, poor diet, and physical inactivity are prevalent among adolescents in the United States and are major contributors to preventable morbidity and mortality (1). These behaviors continue into adulthood (2) and increase the risk of heart disease, osteoporosis, stroke, certain cancers, and other chronic diseases (1). National surveys (3) indicate that 8% of middle school students and 23% of high school students used cigarettes on one or more of the past 30 days. Nine percent of high school students report frequent, current cigarette smoking on 20 or more of the past 30 days (4). Survey data further indicate that the majority of adolescents do not consume diets that meet the Dietary Guidelines for Americans 2005 (http://www.health.gov/dietaryguidelines/dga2005/document/), and many young people are physically inactive (1,5).

An inadequate diet and physical inactivity may compound the deleterious effects of smoking on health. For example, some research indicates that adolescents who smoke may be less likely to exercise or to consume adequate amounts of calcium-rich foods and beverages (6). As smoking cigarettes, inactivity, and poor dietary intake of calcium during adolescence are independent risk factors for osteoporosis, people who begin smoking during adolescence may be at particular risk for osteoporosis later in life (7). Smokers are at increased risk for atherosclerosis and heart disease because of substances in cigarette smoke that promote the formation of free radicals and plaques (8). Vitamin C and other antioxidants neutralize free radicals and may help to prevent oxidative damage. Because of the effect of smoking on free-radical formation, smokers have higher metabolic requirements for vitamin C; therefore, the negative health impact of poor or marginal diets is greater for smokers than for nonsmokers. Observational studies examining patterns of health behavior covariation in adolescents have related smoking behavior to irregular meal patterns (9), greater intakes of soft drinks (10), and lower intakes of fruits, vegetables, and dairy foods (6,11). In addition, findings from cross-sectional and longitudinal research suggest an inverse relationship between physical activity and cigarette smoking during adolescence (6,12).

Although previous studies have reported covariation of smoking behavior with other health behaviors, little research has examined how smoking may be related to dietary intake of key nutrients (e.g., calcium, vitamin C, iron), frequency of fast-food meals, sedentary lifestyle behaviors, or weight status. Furthermore, few studies have been able to consider the association between smoking and a comprehensive range of behaviors within a single population of adolescents. A clear understanding of the interrelationships between these behaviors would help to inform future interventions, such as coordinated school health programs (CSHPs). CSHPs combine nutrition education, physical education, health education, and health services in a climate that supports healthy lifestyle choices in order to promote and maintain the well-being of students (13). Such programs are ideally suited to addressing the intersection of risk behaviors, teaching healthy lifestyle skills, and developing self-efficacy to improve or maintain one's own health.

Our study built on previous research by examining how smoking is related to physical activity patterns, eating behaviors, and diet within a diverse, population-based sample of adolescents in Minnesota. The specific research questions we addressed were the following:

1) Are patterns of physical activity (vigorous and moderate activity and participation in team sports) and sedentary activity (watching television and videos) related to cigarette smoking among adolescents?

2) Are eating behaviors (frequency of eating breakfast, lunch, dinner, family meals, and fast-food meals) related to smoking frequency?

3) Is diet quality (intake of foods and nutrients) related to cigarette smoking among adolescents?

The study's hypothesis was that healthy behaviors (i.e., vigorous and moderate physical activity; participation in team sports; eating regular meals; participation in family meals; and consumption of fruits, vegetables, grains, fiber, and micronutrients) would be inversely related to frequency of cigarette smoking and that unhealthy behaviors (i.e., sedentary activity; fast-food meals; and intakes of fat, soft drinks, and caffeine) would be directly related to smoking frequency.

## Methods

### Study design

Data for this analysis were drawn from Project EAT (Eating Among Teens), a study designed to investigate socioenvironmental, personal, and behavioral correlates of dietary intake among adolescents aged 11 through 18 years (14). (In the context of this study, we define adolescents as young people aged 11 through 18 years.) Following approval of the study by the University of Minnesota Human Subjects' Committee and by the research boards of participating school districts, a trained staff collected survey and anthropometric data in health, physical education, and science classrooms during the 1998–1999 school year. Students completed surveys and had their height and weight measured within a private area of the school. Student assessments included the Project EAT survey and the Youth and Adolescent Food Frequency Questionnaire (YAQ), which measures usual dietary intake. Additional details about the development of the Project EAT survey can be found in previous publications (15).

### Study sample

The overall study sample of students who completed the Project EAT survey (N = 4746 adolescents, response rate = 81.5%) was ethnically and socioeconomically diverse. Students aged 11 through 18 years from 31 public middle and high schools in the Minneapolis-St. Paul area participated in the study. Student participants were equally divided by sex, with 2377 (50.1%) males and 2357 (49.7%) females (12 students [0.2%] did not indicate their sex). The mean age of students in the study sample was 14.9 years (range 11 to 18 years); 33.9% (1608 students) were in middle school, and 64.8% (3074 students) were in high school (64 students [1.3%] did not indicate their grade). The racial and ethnic backgrounds of study participants were as follows: 2264 (47.7%) white, 887 (18.7%) African American, 896 (18.9%) Asian American, 273 (5.8%) Hispanic, 165 (3.5%) Native American, and 186 (3.9%) mixed or other (75 students [1.5%] did not indicate their background).

### Measures

#### Demographics and weight status

Study participants self-reported sex, race and ethnicity, and socioeconomic status on the Project EAT survey. Race and ethnicity were assessed with the following question: "Do you think of yourself as (a) white, (b) black or African American, (c) Hispanic, (d) Asian American, (e) Hawaiian or Pacific Islander, or (f) American Indian or Native American." Subjects could choose more than one category; those responses indicating multiple categories were coded as *mixed or other*. Because there were few participants who identified themselves as Hawaiians or Pacific Islanders, these participants were also included in the *mixed or other* category.

Classification tree methodology (16) was used to generate five categories of socioeconomic status (SES). The prime determinant of SES was parental educational level, defined by the higher level of either parent. Secondary variables were family eligibility for public assistance, student eligibility for free or reduced-cost school meals, and parental employment status (14).

Trained research staff measured heights and weights using standardized equipment and procedures. Body mass index (BMI) was calculated by dividing weight in kilograms by the square of height in meters (i.e., [weight in kilograms]/[height in meters]^2^). Respondents were classified according to sex- and age-specific cut-off points as not overweight (BMI <85th percentile), moderately overweight (BMI ≥85th to <95th percentile), or very overweight (BMI ≥95th percentile).

#### Behaviors

Study participants self-reported smoking, physical activity, and sedentary activity on the Project EAT survey. A single item modified from the Minnesota Adolescent Health Survey assessed how often students had smoked cigarettes during the past year (17). Response categories were *never*, *a few times*, *monthly*, *weekly*, or *daily*. On the basis of self-reported smoking frequency, adolescents were then categorized into one of three groups roughly corresponding to the categories employed in analyses of surveys from the Youth Risk Behavior Surveillance System (YRBSS) (5): 1) *nonsmokers* reported never smoking or smoking only a few times, 2) *current smokers* reported smoking at least monthly but less often than once per day, and 3) *daily smokers* reported smoking daily during the past year.

A modified version of the Leisure Time Exercise Questionnaire was used to measure physical activity (18). Students were asked to report how many hours they spent during a usual week in strenuous exercises (e.g., biking fast, aerobic dancing, running, jogging, swimming laps, rollerblading, skating, lacrosse, tennis, cross-country skiing, soccer, basketball, football) and moderate exercises (e.g., walking quickly, baseball, gymnastics, easy bicycling, volleyball, skiing, dancing, skateboarding, snowboarding). Response options for these questions were 0, <0.5, 0.5 to 2, 2.5 to 4, 4.5 to 6, and >6 hours per week and were recoded to 0, 0.3, 1.3, 3.3, 5.3, and 8 hours per week. Total hours of vigorous physical activity and moderate physical activity per week were calculated by summing the recoded values.

As a measure of sedentary behavior, students were asked to report the average number of hours they watched television and videos on weekdays and on weekends. Response options for these questions were 0, 0.5, 1, 2, 3, 4, and ≥5 hours. An average number of hours spent watching television and videos per week was computed from responses. A single item adapted from the YRBSS asked students to indicate the number of sports teams (0, 1, 2, or ≥3) they played on during the past year (19). Participation in team sports was defined as playing on one or more teams. Pretesting of physical activity and inactivity measures in a sample of 161 adolescents found test-retest reliability coefficients of *r* = .63 for vigorous physical activity, *r* = .52 for moderate physical activity, *r* = .81 for weekday television and video watching, and *r* = .69 for weekend television and video watching.

Eating behaviors assessed were dietary intake, eating breakfast, eating lunch, eating dinner, participating in family meals, and consuming fast-food meals. We employed the 149-item YAQ to assess dietary intakes of total energy (kcals), fruits and vegetables (servings), grains (servings), soft drinks (servings), total fat (% of total energy), fiber (g), calcium (mg), iron (mg), zinc (mg), vitamin A (IU), vitamin C (mg), folate (mcg), and caffeine (mg). Other research has documented the validity and reliability of the YAQ for use in adolescents (20,21). Among a sample of adolescents aged 9 to 18 years, the mean correlation between energy-adjusted nutrient intakes assessed with the YAQ and with three 24-hour recalls was 0.45 (20). The mean intake of energy as assessed by the YAQ was higher than for recalls but within 1% (20). Reproducibility coefficients for responses on two YAQs administered one year apart were 0.48 for fruits and vegetables, 0.48 for grains, and 0.57 for soft drinks (21). Self-report measures assessing past-week frequency of eating breakfast, lunch, and dinner; participating in family meals; and consuming meals from a fast-food restaurant were included on the Project EAT survey (22). Response options ranged from *never* to *every day* or *more than 7 times*.

### Statistical analyses

Descriptive statistics were calculated to examine the association of frequency of smoking with demographic characteristics in our sample. We used chi-square tests to test bivariate associations between smoking and characteristics of adolescents.

We used mixed model linear regression, controlling for sex, grade level (middle school or high school), race and ethnicity (in six categories), and SES (in five categories) on physical activity and eating behaviors to generate adjusted prevalences by smoking status. Outcomes were dichotomized: meeting recommendations for moderate physical activity (30 minutes on ≥5 days or ≥2.5 hours per week), meeting recommendations for vigorous physical activity (20 minutes on ≥3 days or ≥1 hour per week), participating in team sports (on at least one team), exceeding the maximum recommended for hours of television or video watching (≥14 hours per week), eating regular meals (eating breakfast, lunch, and dinner 5–7 days per week), participating regularly in family meals (5–7 days per week), and frequently eating fast-food meals (≥3 meals per week). School was included in the models as a random effect to control for possible intracluster correlation of responses from students in the same school. Identical models but with the logistic link function and binomial error were used to generate *P* values for testing differences in adjusted prevalences over the three categories of smoking status.

We used the same mixed model linear regression described above with total energy as an added covariate to generate adjusted mean dietary intakes of adolescents by smoking status and *P* values for testing differences. An adjustment for total energy intake was applied to examine differences in diet quality between smokers and nonsmokers because the average total energy intake of smokers was greater than the average energy intake of nonsmokers (23). For dietary outcomes that exhibited positive skewness, identical models but with the square-root transformation were used to generate *P* values. A 95% confidence level was used to interpret the statistical significance of probability tests. All analyses were conducted using the Statistical Analysis System (SAS), version 8.2 (SAS Institute, Cary, NC, 2001).

## Results

### Characteristics of adolescents who smoke cigarettes

In the study sample, nearly 7% of adolescents reported smoking at least monthly but less frequently than once per day, and another 10.3% reported smoking daily (Table 1). Middle school students reported lower smoking rates than high school students. The percentage of high school students smoking at least monthly, 22.1% (639/2887 students), was nearly three times higher than that of middle school students smoking as often, 7.4% (109/1477 students). (Denominators were based on the total numbers in high school and middle school, respectively, that responded to both items assessing cigarette use and grade level. These numbers match the denominators in the first column of Table 1.) Rates of smoking were lowest among African American and Asian students. More young people of middle and low-middle SES reported frequent smoking than did those of low SES or high SES. Smoking frequency was similar among males and females, and no significant differences in smoking frequency were observed according to weight status based on BMI.

### Physical activity and eating behaviors by smoking status

Table 2 presents adjusted prevalences of physical activity and eating behaviors according to smoking status. Participation in team sports was significantly and inversely related to smoking frequency. Of 457 students who reported daily smoking, only 46.0% had played on a sports team during the past year compared with 64.5% of 3657 nonsmokers. We observed no significant differences between frequent smokers and nonsmokers for moderate physical activity or hours of television and video watching. However, differences in the observed prevalences of participants meeting the recommendation for vigorous physical activity according to smoking status were at the cut-point of statistical significance (*P* = .05). A higher percentage of nonsmokers (79.6% of 3657 students) met the recommendation for participation in weekly vigorous physical activity compared with students who reported daily smoking (71.0% of 457).

Different eating patterns were also observed among adolescents who reported more frequent smoking. Smoking frequency was significantly and inversely related to regularly eating breakfast, lunch, and dinner. Frequent consumption of fast-food meals was significantly and directly related to smoking frequency. More than one third of daily smokers reported three or more fast-food meals per week while fewer than one fifth of nonsmokers reported this number. An inverse association of borderline statistical significance (*P* = .06) was found between participation in family meals and adolescent smoking frequency.

### Diet Quality by Smoking Status

Table 3 presents means of daily energy intake and dietary intake of foods and nutrients adjusted for energy intake according to smoking status. Smoking was associated with higher energy intake: daily smokers reported mean intakes of an additional 264 kcals per day compared with nonsmokers. Before adjustment of food and nutrient intake for total daily energy intake (data not shown), smoking was associated with higher intakes of grains, soft drinks, and caffeine and lower intakes of vitamin A. After adjusting for total energy intake, greater smoking frequency was associated with several indicators of a lower-quality diet. Consumption of fruits and vegetables, grains, fiber, calcium, iron, zinc, vitamin A, vitamin C, and folate were significantly and inversely related to smoking frequency. Intakes of soft drinks and caffeine were significantly and directly related to smoking frequency.

Because of the substantially lower rates of smoking among middle school versus high school students and differences between the dietary habits of male and female adolescents in our sample, interactions were tested to assess for effect modification by grade level or sex. No statistically significant interactions were identified and thus are not discussed further.

## Discussion

This study described cigarette smoking behaviors among a diverse sample of middle and high school students and the association of cigarette smoking status with a range of lifestyle behaviors, including physical activity, eating patterns, and dietary intake. Self-reported current (past month) cigarette smoking rates in the sample were similar but somewhat lower than rates reported by national surveys of middle school students (7% versus 9%) and high school students (22% versus 28%) in 1999 (24). In the present study, smoking was associated with grade level (high school), race and ethnicity (i.e., Native American, white, Hispanic), and with low-middle and middle SES but not with sex or weight status. These findings about the relationship of smoking with sex, race and ethnicity, and grade level are consistent with national surveillance data (3). However, the lack of a relationship with weight status was not consistent with other findings that have shown higher rates of smoking among overweight adolescents (25).

Overall, we found that adolescents who smoke were less likely to have healthful eating and physical activity habits. In agreement with other research, smoking frequency was inversely related to team sport participation (26), but we did not observe strong associations with moderate or vigorous physical activity. Other research studies have reported strong inverse associations between physical activity and smoking, and it is possible that our findings did not support these studies because of measurement differences (6,12). Most other studies have considered frequency rather than total hours of weekly physical activity and have focused only on vigorous physical activity that makes one breathe hard (6,12). Our study also found an association suggesting reduced vigorous physical activity in adolescents who smoke more frequently; however, this association was at the cut-off point of statistical significance (*P* = .05). Observations indicating a potential inverse association between smoking frequency and vigorous physical activity in adolescents are of particular concern because of the tendency for physical activity to decline as adolescents transition to young adulthood and because research has linked declining activity levels to increases in BMI (27,28).

Because few studies have examined associations between smoking frequency and sedentary behaviors, in this study we considered whether a relationship exists between smoking and hours of watching television or videos. Although we found that the prevalence of exceeding the maximum recommended for hours of television- and video-watching was high in each category of smoking, exceeding the recommendation was unrelated to smoking frequency. These results contradicted our hypothesis, which we based on a prospective study of media use and smoking initiation in young people (29). Given the observation in previous research of a direct, dose-response relationship between television viewing hours and rates of smoking initiation (29), we hypothesized that more frequent smoking would be directly related to time spent watching television and videos. Although use of these media may be related to smoking initiation, in this study it did not appear to be related to the frequency of smoking among adolescents.

We observed associations between smoking and eating behaviors with greater consistency than associations between smoking and physical activity. One strong finding among students in our study was a direct association of smoking frequency with irregular meal patterns at breakfast, lunch, and dinner. Irregular meal patterns have been related to poorer intakes of key micronutrients (e.g., calcium, zinc, vitamin C, iron) and greater consumption of sugar (30). Few other studies have investigated whether smoking frequency is related to fast-food consumption or family meal patterns (31,32). The results of our study were in agreement with another study, which indicated that adolescent smoking frequency is directly related to more frequent intake of fast food (32). In a previous analysis of the Project EAT data, we found associations between participation in family meals and a number of substance-use behaviors, including smoking (31). Frequent intake of fast-food meals and irregular family meal patterns are of concern as these behaviors have also been related to diets of lower nutritional quality in adolescents and because fast-food intake has been shown to prospectively predict risk of increasing BMI in adolescent females (33-35).

To investigate relationships between smoking and diet quality, we examined whether smoking frequency is associated with food-group servings and intake of selected nutrients among adolescents. In general, smoking frequency was inversely related to healthful food selections and to nutrient intakes adjusted for total energy intake. These findings build on previous studies that have reported similar associations between food choices and smoking behavior in adolescents (6,36). The more comprehensive assessment of dietary patterns in this study demonstrated that, compared with the diets of nonsmokers, the diets of adolescents who reported frequent smoking were lower in several key nutrients important for their roles in the prevention of chronic disease (e.g., calcium for the prevention of osteoporosis).

One of the strengths of this study was its large and diverse sample of adolescents, which allowed us to assess for the effect of modification by sex and grade level. Other strengths were the range of physical activity and eating behaviors evaluated within this sample and the comprehensive assessment of dietary intake accomplished using a validated instrument (YAQ) (20). The response rate of 81.5% was relatively high, and the demographic makeup of the study sample approximated the makeup of the source school populations.

In interpreting the results of this study, certain limitations should also be considered that may have interfered with our ability to observe the total effect of associations between smoking, physical activity, and nutrition behaviors. For example, parents' level of education is a widely used indicator of SES, but the economic benefits of higher education are not uniform, and this indicator may have produced incomplete adjustment for SES (37). Despite efforts to further adjust associations for sex, grade level, and race and ethnicity, it is also possible that factors other than the covariates included in our models are related to the associations between smoking and patterns of poor diet and physical activity. This also could have influenced the observed pattern of associations.

Finally, using only a single item to assess cigarette use may have led to some misclassification of smoking status. Misclassification would have attenuated associations of smoking with physical and sedentary activity, dietary patterns, and BMI. Additional measures of lifetime smoking habits would have allowed for analyses to consider whether established habitual patterns of smoking behavior are associated with unhealthy lifestyle behaviors. Future research should use stronger measures of smoking behavior and use longitudinal study designs to clarify the temporal order of increases in smoking and decreases in healthful eating and activity behaviors.

Collectively, this study and previous research suggest the importance of early intervention in adolescents on smoking, physical activity, and dietary patterns. It is possible that the observed covariation in health behaviors is due to a greater belief in chance as an influence on health and a lower perceived benefit of engaging in healthful behaviors (38,39). Researchers are still learning how adolescents receive, process, and prioritize information about health risks and how they modify their behavior as a result. Therefore, young people who smoke may benefit from comprehensive, coordinated interventions that address self-efficacy to improve or maintain personal health by engaging in regular physical activity, healthful eating behaviors, and other positive lifestyle behaviors in addition to reducing cigarette smoking. CSHPs are one example of how messages may be combined to address and reinforce multiple lifestyle behaviors through classroom instruction and environmental interventions (40). These programs can involve all areas of a school in promoting good nutritional habits, physical activity, and a nonsmoking lifestyle. Programs should be designed not only to teach adolescents about the benefits of engaging in healthful behaviors but also the behavioral skills necessary to carry them out (40). In addition, the school environment should be supportive of healthy lifestyle behaviors (e.g., healthy food available in the cafeteria and in vending machines, teachers and staff serving as role models for a physically active, smoke-free lifestyle). To have the greatest impact on reducing risk for chronic disease in adulthood, prevention programs need to target those at highest risk for unhealthy behaviors, engage youth early in adolescence, and focus on specific behaviors so that young people can observe and learn the benefits of adopting healthful lifestyle behaviors.

## Figures and Tables

**Table 1:** Cigarette Smoking Status of Minnesota Adolescents, by Characteristics, 1998–1999

Characteristic	n^a^	Frequency of Smoking Cigarettes			*P* Value

		Nonsmokers, %	Current Smokers^b^, %	Daily Smokers, %	
Total	4413	82.9	6.8	10.3	Does not apply
Sex					
Male	2213	83.5	6.6	9.9	.46
Female	2199	82.2	7.0	10.8	
Grade level					
Middle school	1477	92.6	4.7	2.7	<.001
High school	2887	77.9	7.9	14.2	
Race^c^					
African American	735	90.9	4.6	4.5	<.001
Asian American	843	88.1	6.2	5.7	
Hispanic	243	80.7	8.2	11.1	
Native American	156	69.2	6.4	24.4	
White	2209	79.6	7.5	12.9	
Mixed or other	168	80.3	7.8	11.9	
SES^d^					
Low	738	83.9	6.6	9.5	<.001
Low-middle	803	78.5	6.3	15.2	
Middle	1142	82.4	6.4	11.2	
Upper-middle	1019	84.1	8.3	7.6	
High	609	84.9	5.9	9.2	
Weight Status					
Not overweight	2753	83.0	6.4	10.6	0.21
Overweight	697	82.1	8.3	9.6	
Very overweight	581	83.2	7.9	8.9	

SES indicates socioeconomic status.

a The sample size for different variables may vary from the total sample size because of missing responses. There were 333 adolescents who did not respond to the survey item that was used to assess frequency of smoking cigarettes.

b At least monthly but less often than daily smoking.

c Participants could chose more than one category; responses indicating multiple categories were coded as *mixed or other*.

d The prime determinant of SES was the higher educational level of either parent. Subsidiary variables were family eligibility for public assistance, student eligibility for free or reduced-cost school meals, and parental employment status.

**Table 2:** Percentages of Minnesota Adolescents Engaging in Physical Activity, Sedentary Activities, and Eating Behaviors by Cigarette Smoking Status, 1998–1999

Behavior (Criterion)	Smoking Status^a^			

	Nonsmokers % (95 % CI) n^b^ = 3657	Current Smokers^c^% (95% CI) n^b^ = 299	Daily Smokers % (95% CI) n^b^ = 457	*P *Value^c^
Engage in moderate physical activity (≥2.5 hours per week)	52.9 (50.3-55.5)	53.0 (47.0-59.0)	46.9 (41.7-52.1)	.18
Engage in vigorous physical activity (≥1 hour per week)	79.6 (77.0-82.2)	74.4 (69.2-79.6)	71.0 (66.6-75.4)	.05
Participate in team sport (≥1 teams)	64.5 (62.7-66.3)	61.2 (55.6-66.8)	46.0 (41.4-50.6)	.01
Watch television or video (≥14 hours per week)	93.5 (92.5-94.5)	91.6 (88.6-94.6)	92.8 (90.4-95.2)	.51
Eat breakfast (5-7 days per week)	48.3 (45.1-51.5)	31.8 (25.6-38.0)	29.1 (23.7-34.5)	.01
Eat lunch (5-7 days per week)	78.0 (76.0-80.0)	69.6 (64.6-74.6)	60.5 (56.1-64.9)	.005
Eat dinner (5-7 days per week)	86.6 (85.0-88.2)	80.8 (76.6-85.0)	69.2 (65.6-72.8)	.02
Participate in family meals (≥5 meals per week)	47.4 (45.0-49.8)	36.7 (30.7-42.7)	32.0 (27.0-37.0)	.06
Eat fast food (≥3 meals per week)	19.7 (17.5-21.9)	28.9 (23.9-33.9)	36.6 (32.2-41.0)	.03

Nonsmokers indicate those who reported never smoking or smoking only a few times; current smokers, those who reported smoking at least monthly but less often than once per day; daily smokers, those who reported smoking daily during the past year.

a Prevalences of performing physical activity and engaging in eating behaviors according to a specified criterion are adjusted for sex, grade level (middle versus high school), race and ethnicity, and socioeconomic status. School was included in models as a random effect.

b The sample size for different behaviors may vary from the total sample size because of missing responses for the variable and covariates.

c Represents testing for differences in adjusted prevalences over the three categories of smoking status (2 *df*).

**Table 3:** Mean Daily Dietary Intake Among Minnesota Adolescents by Cigarette Smoking Status, 1998–1999

Dietary Intake	Smoking Status^a^			

	Nonsmokers Mean (SE) n^b^ = 3358	Current Smokers^c^ Mean (SE) n^b^ = 276	Daily Smokers Mean (SE) n^b^ = 411	*P *Value^c^
Total energy (kcal)	2104 (42)	2306 (74)	2368 (65)	<.001
Fruits and vegetables (servings)	4.1 (0.07)	3.8 (0.15)	3.5 (0.13)	<.001
Grains (servings)	6.0 (0.05)	5.7 (0.12)	5.8 (0.10)	.006
Soft drinks (servings)	1.29 (0.03)	1.49 (0.06)	1.76 (0.05)	<.001
Total fat (% kcal)	29.9 (0.12)	30.4 (0.33)	29.7 (0.28)	.29
Fiber (g)	17.0 (0.16)	16.3 (0.33)	15.4 (0.29)	<.001
Calcium (mg)	1118 (12)	1077 (25)	1060 (21)	.004
Iron (mg)	13.9 (0.09)	12.8 (0.23)	12.5 (0.20)	<.001
Zinc (mg)	10.9 (0.05)	10.3 (0.15)	10.3 (0.13)	<.001
Vitamin A (IU)	8774 (143)	8164 (318)	7074 (274)	<.001
Vitamin C (mg)	151 (1.7)	138 (4.6)	142 (3.9)	.001
Folate (mcg)	295 (2.2)	272 (5.6)	268 (4.8)	<.001
Caffeine (mg)	41 (0.7)	60 (2.4)	68 (2.0)	<.001

Nonsmokers indicate those who reported never smoking or smoking only a few times; current smokers, those who reported smoking at least monthly but less often than once per day; daily smokers, those who reported smoking daily during the past year. SE indicates standard error.

a Means adjusted for sex, grade level (middle versus high school), race and ethnicity, and socioeconomic status. Models for foods and nutrients additionally adjusted for total energy intake. School was included in models as a random effect.

b Numbers are reduced from the total sample because of 1) missing covariates needed for adjustment and 2) missing or implausible responses to the food frequency questionnaire. Of the total sample, 344 participants did not complete the food frequency questionnaire and 258 participants were excluded because they provided biologically implausible responses (defined a priori as having energy intakes below 400 kcal/day or over 7,000 kcal/day).

c Represents testing for differences in adjusted means over the three categories of smoking status (2 *df*).
